# Faster clinical response to the onset of adverse events: A wearable metacognitive attention aid for nurse triage of clinical alarms

**DOI:** 10.1371/journal.pone.0197157

**Published:** 2018-05-16

**Authors:** Daniel C. McFarlane, Alexa K. Doig, James A. Agutter, Lara M. Brewer, Noah D. Syroid, Ranjeev Mittu

**Affiliations:** 1 Patient Care & Monitoring Solutions, Innovation Office, Royal Philips, Andover, Massachusetts, United States of America; 2 College of Nursing, University of Utah, Salt Lake City, Utah, United States of America; 3 College of Architecture & Planning, University of Utah, Salt Lake City, Utah, United States of America; 4 Applied Medical Visualizations (Medvis), Salt Lake City, Utah, United States of America; 5 Department of Anesthesiology, University of Utah Health Sciences Center, Salt Lake City, Utah, United States of America; 6 Anesthesiology Center for Patient Simulation, University of Utah, Salt Lake City, Utah, United States of America; 7 Information Technology Division, Information Management and Decision Architectures Branch, United States Naval Research Laboratory, Washington, District of Columbia, United States of America; University Antwerp, BELGIUM

## Abstract

**Objective:**

This study evaluates the potential for improving patient safety by introducing a metacognitive attention aid that enables clinicians to more easily access and use existing alarm/alert information. It is hypothesized that this introduction will enable clinicians to easily triage alarm/alert events and quickly recognize emergent opportunities to adapt care delivery. The resulting faster response to clinically important alarms/alerts has the potential to prevent adverse events and reduce healthcare costs.

**Materials and methods:**

A randomized within-subjects single-factor clinical experiment was conducted in a high-fidelity 20-bed simulated acute care hospital unit. Sixteen registered nurses, four at a time, cared for five simulated patients each. A two-part highly realistic clinical scenario was used that included representative: tasking; information; and alarms/alerts. The treatment condition introduced an integrated wearable attention aid that leveraged metacognition methods from proven military systems. The primary metric was time for nurses to respond to important alarms/alerts.

**Results:**

Use of the wearable attention aid resulted in a median relative within-subject improvement for individual nurses of 118% (W = 183, p = 0.006). The top quarter of relative improvement was 3,303% faster (mean; 17.76 minutes reduced to 1.33). For all unit sessions, there was an overall 148% median faster response time to important alarms (8.12 minutes reduced to 3.27; U = 2.401, p = 0.016), with 153% median improvement in consistency across nurses (F = 11.670, p = 0.001).

**Discussion and conclusion:**

Existing device-centric alarm/alert notification solutions can require too much time and effort for nurses to access and understand. As a result, nurses may ignore alarms/alerts as they focus on other important work. There has been extensive research on reducing alarm frequency in healthcare. However, alarm safety remains a top problem. Empirical observations reported here highlight the potential of improving patient safety by supporting the meta-work of checking alarms.

## Introduction

The United States (U.S.) spends more per person on healthcare than any other country [1]. Medical errors in hospitals, however, are the third leading cause of death in the U.S. (on average 602–689 error-related deaths per day) [2]. Additionally, adverse events (permanent or temporary harm) currently occur in 27–33% of all U.S. hospital admissions. Therefore, of the about 96,058 total admissions per day [3], about 25,935–31,699 will experience at least one adverse event [4,5], 44% of which are "clearly or likely preventable" ([4], p. 22). In addition to causing patients harm or death, adverse events are also a primary cause of uncontrolled variation in healthcare costs (adding $334-$75,000 per case [6–8]) and break the financial predictability needed for an outcome-based healthcare model [9].

In the seminal report "To Err is Human: Building a Safer Health System," The Institute of Medicine emphasizes that most medical errors are caused by quality problems with healthcare systems, not by reckless individuals. "The focus must shift from blaming individuals for past errors to a focus on preventing future errors by designing safety into the system" ([10], p. 5). One study of intensive care unit (ICU) nurses shows that nurses are typically aware of multiple types of healthcare system design problems or "performance obstacles" that cause unsafe conditions [11].

To mitigate the risk of errors, the design of a quality healthcare system must address the important human aspects of communication and care coordination for healthcare delivery. Electronic medical record (EMR) systems can improve control over the costs of delivering effective clinical care relative to patients’ existing care plans [12,13]. However, patients also require ongoing surveillance, dynamic care coordination, verification of care delivery, and revision of care plans to match their changing needs [14–17]. To keep patients safe, clinicians must maintain constant awareness of patients’ changing needs and dynamically adapt care delivery to mitigate risks [14]. Usability problems with information services block this awareness by overwhelming users (i.e., information overload) [18] and can cause clinicians to miss opportunities to correct emerging problems that lead to adverse events. These additional meta-level activities for clinical ’communications and care coordination’ are required to maintain the validity of patients’ care plans over time.

The U.S. Centers for Medicare & Medicaid Services’ (CMS) National Quality Strategy (NQS) Domains highlights ’Communications and Care Coordination’ as essential for quality healthcare [19]. Observation studies describe the complex issues for effective handoff communication [20–22]. Other qualitative studies show that during human-human interruption among nurses that both the interrupter and the interruptee use contextual information to broker an interruption for starting a conversation either in-person [23] or by mobile communication device [24,25]. Observation studies show that nurses participate in the work and meta-work of coordinating recovery from errors [26]. They typically monitor their own performance and changing work context, and when errors are detected they address the problem and try to find ways to prevent them in the future.

Clinical staff, including nurses, are often fully engaged in delivering preplanned care such as medication administration, completing medical or nursing procedures, risk assessment and injury prevention, and patient education [27]. This high workload includes heavy multitasking and frequent interruptions [28,29] that can cause distraction [30–32]. Because they have committed their full cognitive resources to delivering ’effective clinical care,’ it is difficult for clinicians to also find sufficient time to concurrently perform the crucial meta-level tasks of: surveillance, care coordination, care delivery verification, and revision of care plans [33]. As a result, clinical performance on the CMS-emphasized ’communications and care coordination’ tasks is often poor [34].

It also explains the common problem of alarm fatigue where alarm/alert signals are ignored altogether [35]. The high cognitive workload and lack of access to alarm/alert-based information create a problem where nurses lose track of patients’ evolving situations [36]. This shortfall is a root cause of adverse events because care delivered to plan is ineffective once the plan becomes invalid. Poor surveillance leads to failure to rescue from adverse physiological changes [37,38]. Poor coordination leads to missed nursing care (errors of omission) [39,40] or well-intentioned delivery of care that does not match needs (a hidden type of error of commission). Poor verification leads to medical errors that go unrecognized [41,42]. Poor revision of care plans leads to neglect or lack of attention to new care needs. For example, if a nurse recognizes that a patient may be septic, but doesn’t notify the healthcare team or initiate a sepsis bundle (i.e., goal-directed therapy), then the patient will likely continue to deteriorate [38].

The research reported here explores novel technologies to improve ’communications and care coordination’ and showcase the unrecognized high-potential for this topic to improve the quality of healthcare. It also addresses the current shortfall in quantitative experimental research approaches to explore and improve the quality of systems to support these issues.

## Background

Recognizing unexpected needs for coordination, verification, and replanning requires constant vigilance across a dense information landscape [43,44]. However, research in human factors across many different fields shows that people are generally poor at sustained attention and vigilance tasks [38,45–47]. Most patient adverse events are preceded by observable warning signs that could potentially be automatically detected and announced to care givers as alarms/alerts [44]. Monitoring technologies can automatically detect these changes and have the potential to enable clinicians to more quickly recognize and address patient problems and prevent adverse events [48]. Modern automated vigilance technologies for monitoring and generation of alarm signals are technically mature and scalable to high-volume streaming data [35,49,50]. However, predicting an adverse event is usually more complex than the usual single-parameter alerting mechanisms in current fielded use [51]. The information needed by clinicians to understand and triage patient change is often distributed across multiple different sources [36,52–55].

### Alarms & alerts—An approach with unrealized potential

Automatically detecting patterns in data and generating alarm/alert signals is only one aspect of an alarm system. There is also an ’air gap’ between the automation and the human users that must be bridged. For automated monitoring to be useful, the generated alarm/alert signals must achieve conscious situational awareness within a clinician user. From a patient’s perspective, an important actionable alarm/alert signal that fails to cause this conscious awareness for a clinician is no different than a highly dangerous false negative. Unfortunately, alarms/alerts frequently fail to bridge this gap and their potential to prevent adverse events is not realized in practice. Alarm/alert signals are often ignored by clinicians, and alarm safety is cited as a "number one" patient safety problem [56,57]. Prior research in this area has mainly focused on the persistent problems of high alarm/alert rate (96–350 per bed per day) and the high percentage of false or clinically non-actionable alarms (80–99.4%) [35,58–61].

Table 1 summarizes eight different approaches being explored to solve the continuing alarm safety crisis. Research has shown the potential to reduce some frequency of false or non-actionable alarms/alerts through (see Table 1A and 1B): re-configuration of the alarm parameter limits [60,62–64]; alarm escalation [65], individualizing alarm configuration for each patient [66]; improved leads connection [67,68]; and policies for integrated ’middleware’ data environments [69,70]; Other studies show advancement of integrated multi-parameter algorithms [71–75]. This focus over the last three decades on reducing alarm frequency [76,77], however, has been criticized as being largely ineffective [78], with only a couple exceptions [48,79]. Fully-automated approaches have challenging side-effects that include (see Table 1G): user trust of automation; high degree of expert labor required for complex use and configuration; and the risk of introducing false negatives.

**Table 1:** Approaches for solving the alarm/alert safety crisis.

Approach	Pros	Cons	Methods	Status
A. Reduce frequency of alarms/alerts [60,62–68]	Minimize frequency of interruptions to clinical workflow	Increased risk of false negatives (the failure to generate an alarm/alert when warranted);	Device configuration settings and policies; best practices; sensor lead management; escalation policies;	Over 3 decades to-date of heavy research investment; multiple hundreds of published articles in the literature; overall, very few lab results translated to hospital practice; multiple legal and regulatory roadblocks have been identified [90]
B. Improve quality of alarms/alerts [69–75]	Minimize amount of clinical time/effort wasted on false or non-actionable interruptions	Increased risk of false negatives;	Middleware to integrate device data; advanced algorithms, incl., multi-parameter algorithms; automated filters;	
C. Constant one-on-one expert nurse attention at a patient’s bedside [91,92]	Minimize dependency on notifications	Extremely high costs of clinical labor per patient	Personal Care Attendant: a dedicated nurse 24/7	Rare because of the extreme high cost; used for delivering intense care in some ICU situations
D. Constant one-on-one non-skilled human attention, with a nurse on-call [91,92]	Reduce dependency on automated notifications	Additional costs of non-clinical labor per patient	Personal care attendant: a ’bed watcher’	Practical application typically limited to a few types of patients
E. Remote brokered triage of alarms/alerts by another experienced nurse or doctor [87,88]	Minimize interruptions and wasted effort for responsible clinicians	Additional costs of clinical labor; errors from fatigue or inattention; and distribution of responsibility for patients	Remote telemetry consoles staffed with clinicians	Practical application typically limited to a few types of patients
F. Remote brokered triage of alarms/alerts by non-clinical person [86]	Reduce interruptions; reduce amount of time wasted on false or non-actionable interruptions	Additional costs of non-clinical labor; errors from fatigue or inattention; and distribution of responsibility for patients	Central stations or remote consoles staffed with monitor technicians;	Practical application typically limited to a few types of patients
G. Closed-loop full-automation [93]	Extremely fast response to change; minimal human labor costs	Increased patient safety risks from consequences of false positives (generation of an alarm/alert when one is not warranted), and false negatives	Automation monitors a patient’s status and dynamically adapts care delivered without human clinical intervention	The pioneering Bionic Pancreas [93] for treating diabetes highlights the potential of this approach; Active R&D investment continues, esp. by military for medical evacuation (MEDEVAC) operations; patient safety problems still under investigation
H. Empower clinicians to more easily use existing alarm alert signals [94] [The focus of this paper]	Minimize meta-work for checking alarms/alerts and engaging nurse insight in triage of changes to patient; no additional labor costs	Wearable devices for nurses must conform to infection control requirements of the healthcare setting	Secondary alarm notification systems; wearable attention-aids for nurses to triage alarms/alerts (this paper)	Relatively little healthcare R&D investment on this topic so far; mature proven solutions exist in other domains (ex., military)

Manual approaches reduce non-actionable alarms through adding clinical labor to triage all alarms (see Table 1C, 1D, 1E and 1F). Lower nurse staffing rates are correlated with more missed care [80] and more adverse events [81–84]. And conversely, more frequent adverse events are positively correlated with a requirement for higher nurse staffing rates to address [85]. Methods for this approach include: central stations staffed with non-clinical monitor technicians [86]; and remote telemetry consoles staffed with actual clinicians [87,88]. Additionally, many hospitals that have central stations do not staff them full-time because of labor cost constraints. Without staffing, central station displays have little value because nurses are typically not within functional visual range [89] and/or don’t attend. Also, central stations typically do not include integrated information from the multiple deployed medical devices, including infusion pumps (IV pumps). Manual challenges associated with central station patient monitoring include: additional staff labor; human monitor fatigue; and distribution of responsibility for patients.

### Comparison of approach options

This research explores the question (approach ’H’ from Table 1): "How can alarm/alert-based information be more easily communicated to clinical users to enable them to assess its meaning in context and act to prevent adverse events?" The main patient safety problem with existing alarm/alert systems may not be too many or poor quality (although these are acknowledged issues), but inadequate support for their consumption in practice.

It might be argued that other approaches have demonstrated more potential than approach ’H’. ’H’, however, has been only lightly treated in the literature and its relative potential is unknown. A very rich and varied set of publications have explored approaches ’A’ and ’B’ (Table 1) and represent a heavy and committed R&D investment in solving the alarm/alert safety crisis. Several hundred papers report the potential technical feasibility of reducing alarm frequency and improving the quality and consistency of alarm generation. However, despite substantial R&D investment for over three decades, results from ’A’ and ’B’ have mostly not transferred into actual fielded use [90]. Alarm safety remains a persistent problem that continues to worsen [95]. The potential for ’A’ and B’ are well demonstrated, but their feasibility for transfer into fielded use seem undetermined.

Results from approaches ’C’, ’D’, ’E’, ’F’, and ’G’ (Table 1) highlight that the most difficult aspect of using existing alarm/alert signals is triaging their clinical meaning in the context of actual patients’ changing situations. The clinical importance of alarms/alerts cannot be pre-determined, but only makes sense in practice within patients’ dynamic clinical context. In other domains, including aircraft cockpit operations [96–99], reports of fatal errors highlight the importance of enabling workers to understand alarms/alerts in context.

Approaches ’C’, ’D’, ’E’, and ’F’ (Table 1) involve an extra human at the patient’s bedside (or virtually at the bedside) to triage alarm/alert signals by proxy for the responsible nurse. This has been proven effective because humans can access the contextual information that automated systems do not (a core challenge for approach ’G’). Except for approach ’C’, these approaches also relieve the responsible nurse from the workload of walking to the relevant bedside to gather the information needed to triage each alarm/alert.

In summary, approaches ’A’ and ’B’ (Table 1) are technically exciting but generally mired in practical concerns related to technology transfer into fielded systems. Approaches ’C’, ’D’, ’E’, and ’F’ are proven and clinically effective (especially ’E’), but restricted in deployment because the costs for additional labor. Approach ’G’ has potential, but has many unanswered patient safety concerns for complex applications. Approach ’H’ is mostly ignored and unrecognized in the healthcare literature and its potential is unknown. In multiple other domains (e.g. military combat systems), however, approach ’H’ is recognized and proven to be very powerful. This paper empirically tests an assertion that this yet-unrecognized approach of attention-aiding for clinicians has high-potential for improving patient safety.

### Leveraging alarm innovation from defense systems

Breakthrough alarm research sponsored by the U.S. Department of Defense has shown that workers’ metacognition is key for the triage of interruption and dynamic coordination of multitasking [49,100]. Metacognition is the meta-level cognition that people use to focus, organize, and regulate their thinking. People have limited cognitive resources for thinking; they also have limited metacognitive resources to organize their thinking [49]. Defense-sponsored interdisciplinary alarm research shows that self-regulation of cognition [101] can be enhanced through external services that facilitate the metacognitive knowledge and processes required to accomplish multiple concurrent activities [102]. This research, called Human Alerting and Interruption Logistics (HAIL). The basic research for HAIL was interdisciplinary and agnostic of application domain. Experiments with human-subjects found that services that support users’ metacognitive activities for dynamic negotiation of multitasking are typically the most useful [49,100,102]. Applied R&D for HAIL created a domain-independent alert mediation engine that was subsequently expanded for specific application with U.S. Navy combat systems.

These novel technologies and methods can be applied to healthcare to explain how to improve performance for CMS-emphasized ’communications and care coordination’ tasks. An analysis of healthcare delivery using metacognition methods shows: (A) dynamic coordination of healthcare is very complicated; and (B) current alarm announcement solutions have an overpoweringly high cognitive workload for clinicians to check the meaning of alarms.

Nurses typically deliver care for multiple patients concurrently. In hospital acute care settings, for example, a registered nurse (RN) will likely be responsible for delivering care to, on average, 4.8 to 6.8 patients at the same time [103]. Since patients’ needs for care delivery are individually different, time-sensitive, and frequently changing (sometimes in important ways), nurses must dynamically intermix the many actions they perform for different patients across time and space [28,29,104,105]. To prevent adverse events, nurses need to be at the right bed at the right time. To accomplish this, each nurse must develop and maintain an internal multitasking schedule of what care tasks he/she will do for which patient, in what location, and in what intermixed sequence.

### High workload to triage alarms/alerts

More than 20 different alarm/alert-generating medical devices are in use in acute inpatient hospital units in U.S. hospitals [106]. Their alarm signals are most commonly not integrated, but delivered independently from each device to clinical users. Types of devices include: physiological monitors and cardiac monitors; infusion pumps; respiratory monitoring equipment; feeding pumps; bed or chair alarms; wound vacuum devices; sequential compression devices; ventilators; and patient call systems.

Triaging an alarm/alert requires the clinician to understand its meaning and relevance to an individual patient’s care [36]. This usually requires substantial effort when the alarm signal is only an audible alarm sounding in a patient’s room, and includes: determining which patient’s alarm/alert is sounding and which RN is responsible; interrupting other tasks; walking to the room; and introduction of other task pressures that interfere with resumption of pre-interruption work [94]; accessing the alarm/alert information; and accessing relevant contextual information (ex., the patient’s vital signs) [107].

Some hospitals have invested in secondary alarm notification systems that send RNs redundant alarm occurrence messages for one or more medical devices [69,108]. These systems can send messages about alarm occurrences by type through a distributed or mobile device: central station, hallway banner, ’computer-on-wheels’ carts (COW) [109], pager [110], wireless phone, or smart phone. Other hospitals have no secondary alarm notification system at all. A few hospitals have implemented sophisticated middleware to collect alarm signals across multiple devices and deliver partially integrated alarm/alert messages [69,70]. At best, relevant clinicians are notified at their mobile location about the types of alarms/alerts that have occurred. However, current solutions do not provide sufficient integrated context information to triage the alarm/alert occurrences without physically visiting the patient [94]. At worst, alarms/alerts noise sounds only at the bedside, and demand triaging from everyone within earshot, including patients themselves and their families [111–113].

The existing high degree of effort to check an alarm/alert overwhelms the potential benefit of all but the most important alarms. It can force clinicians to choose to either perform their planned care delivery or check alarms. The core alarm safety problem, therefore, is not the high alarm rate (an acknowledged annoyance), but rather the high cost in time and mental resources required to determine whether any single alarm is clinically relevant or not [94]. This also clarifies why three decades of advancements in alarm generation technologies have not yet produced a practical solution for care delivery in practice [78,114].

### Problems for improving usability of existing alarm/alert signals

Four problems block progress in enabling nurses to quickly triage alarm/alert signals:

1. Relevant patient information must be accessed across multiple independent devices and other hospital systems [73], and is not organized or summarized to facilitate easy triage of alarm/alert events by clinicians [115];
2. Some of the key types of patient information required to triage alarm/alert events only exist in the human memory of clinicians [116] or on nurses paper ‘brains’ [117] and are not directly accessible by automation;
3. In most healthcare settings, nurses are responsible for multiple patients and must concurrently multitask care delivery tasks across different patients;
4. Nurses are often not in the location where the alarm is sounding and therefore may not receive the signal in a timely manner [89].

Enabling clinicians to better use alarm signals would require a solution that could address all four problems. The set of information needed to triage new alarm/alert signals would have to be dynamically brought together in one place, and then used to make a triage determination. The part of this information that exists across different hospital devices and systems would need to be automatically accessed, combined, and summarized. Nurses would need to be interrupted and asked to dynamically contribute the additional required information from their cognitive memories. Engaging this participation by nurses would also require special support for their mobile work and multitasking. Satisfying all these requirements with a single solution is a very complex design challenge.

### The HAIL-CAT research prototype

A research prototype system for a wearable attention aid was developed to assist in empirically exploring this question of the potential utility of approach ’H’ (Table 1) [94]. It leverages proven design methods from the HAIL work for metacognitive-aiding of alarm triage from military combat systems [49,100,102]. The prototype and the experiment were designed together to highlight the potential utility of approach ’H’ in general. Because of this ambitions goal and the relative novelty of R&D on approach ’H’ for healthcare, the experimental design includes an unusual mix of both extensive control of variables and full scale clinical realism.

This research facilitation is called the HAIL Clinical Alarm Triage (HAIL-CAT) smartwatch [94]. It was designed by an interdisciplinary team to support the dynamic coordination of multitasking with clinical alarms/alerts [94]. HAIL-CAT minimizes nurses’ cognitive effort for triaging new alarms/alerts by integrating multiple separate design ideas proven in other works. These include: a mobile communication device [110], and integrated contextual information [118]. Audio innovation shows mixed results in the healthcare literature [119,120]. Also, literature from aviation safety confirms that design of audio for alarms/alerts is an extremely complex topic that is not fully understood [121], and was therefore out of scope for the HAIL-CAT design. The HAIL-CAT wearable delivers alarm/alert notification directly to nurses’ wrists with sufficient integrated contextual information to support quick-look triage [94]. Nurses can glance at the wearable hands-free and get sufficient information to triage the new alarm/alert. After looking, nurses also have the option to temporarily silenced the new alarm/alert with a button press from the HAIL-CAT smartwatch, or not.

HAIL-CAT alarm announcements include contextual information that engages nurses directly in using their special hands-on perspective to evaluate and triage alarm messages. While certain types of medical errors, such as misreading drug labels, can be prevented with functional constraints [122,123], other more complex errors require ’live’ specific insight and clinical judgement. Generic solutions, like centrally-defined alarm generation/filtration policies, introduce a risk of failure by ignoring key patient-specific insight gained while caring for individual patients [124].

## Methods

A clinical experiment was conducted at the University of Utah, College of Nursing 20-bed patient simulation facility that replicates a full-scale acute care hospital unit. A novel experimental design explored the potential utility for a wearable attention aid to help nurses better use existing alarm/alert signals to recognize the onset of risks of adverse events. Sixteen RNs participated in teams of four; each nurse was responsible for five patients. Nurses worked a high fidelity acute care patient scenario with two parts.

**Hypothesis:** Introduction of a wearable metacognitive attention aid can enable clinicians to easily triage alarm/alert events and more quickly recognize emergent opportunities to adapt care delivery. Faster response to clinically important alarms/alerts has the potential to prevent adverse events and their added healthcare costs.

### Evaluation requirements

The research prototype leverages design methods from outside the healthcare domain to explore ways to enable clinicians to better exploit existing alarm/alert signals. An empirical experiment to assess its potential utility for healthcare must avoid the influences of bias from multiple different sources, including: large variation in performance across nurses; large variation in performance across units and hospitals [125]; large individual differences across patients; and unpredictable onset and non-repeatability of adverse events. An approach using a large-scale clinical trial with such a novel approach could expose human patients to unknown safety risks.

These methods exploit high-fidelity simulation environments that minimize safety risks and afford the implementation of powerful repeated measures experimental designs. Simulation-based R&D approaches shelter human-subjects from exposure to highly-dangerous safety risks (like airplane crashes or exploding missiles). Simulation is also repeatable, and enables within-subjects evaluations that leverage the fact that variance within each individual is much less than variance across different people. Because of superior control over variance, a within-subjects experimental design can achieve the same statistical power as a between-subjects design with only a quarter to an eighth of the number of participants [126]. For example, a repeated-measures experiment with 16 participants can produce results with the same statistical power as a comparable between-subjects design with 64–128 participants.

### Experimental design

A randomized within-subjects single-factor clinical experiment was conducted in a full-scale 20-bed acute care hospital unit simulation. Sixteen RNs, four at a time, cared for five simulated patients each. In the treatment condition, RNs wore a smartwatch-based wearable attention aid prototype (HAIL-CAT leverages proven military metacognition methods). In the control condition, nurses did not wear the smartwatch prototype. Each RN completed both conditions. Condition order was randomized across 4-nurse teams with a balance of two 4-nurse teams doing the control condition first, and two 4-nurse teams doing the treatment condition first (see Table 2). In both conditions, alarm/alert signals were deliver on devices at the patients’ besides.

**Table 2:** Approaches for solving the alarm/alert safety crisis.

Sequence Order	Scenario Part	Experimental Groups: 4-Nurse Teams			
		1	2	3	4
1st	A: ’Morning Meds’	Baseline: No wearable aid	Treatment: Yes wearable aid	Treatment: Yes wearable aid	Baseline: No wearable aid
2nd	B: ’Evening Meds’	Treatment: Yes wearable aid	Baseline: No wearable aid	Baseline: No wearable aid	Treatment: Yes wearable aid

The experiment was designed to test whether the introduction of a wearable attention aid could enable nurses to better triage alarm/alert events and improve dynamic care delivery prioritization. Each nurse performed both parts of a two-part scenario—one part with the smartwatch, and one without. The sequential order for presentation of the two scenario parts was fixed, but the condition order (wearable vs. no wearable) was randomized. Faster response to clinically important alarms/alerts would enable earlier intervention to prevent adverse events. The primary metric was time delay from onset of clinically-important alarms/alerts until the arrival of the nurse at the bedside.

### Human-subjects and setting

The University of Utah Institutional Review Board (IRB) approved this experiment. All participants were informed of their rights for ethical treatment of human-subjects, and voluntarily signed a written informed consent form. Participants were recruited who were registered nurses, at least 21 years of age, and with at least one year of nursing experience in acute care in-patient hospital units. They also could not have been exposed to any of the pilot testing for this project. There was no other categorization of human subjects. This paper does not include any potentially identifying information of participants.

Sixteen RN participants (15 female, 1 male) were organized into four teams of four nurses. The RNs had a median of 6.5 years of experience (range 0.75 to 16 years) and work at representative hospital units across the Salt Lake City region, Utah, United States of America (USA). The simulation setting included 20 beds, each enclosed in a curtained room with a full complement of real hospital equipment and a SimMan-2G mannequin (Laerdal, Wappingers Falls, New York, USA) in a Hill-Rom-1000 hospital bed. In the experiment, nurses used two automated medication and supply cabinets (Omnicell, Mountain View, California (CA), USA) at a central station.

### Clinical scenario for full-unit patient simulation

A two-segment, 180-minute scenario was employed with realistic patient simulations. High rates of patient care data were available from patient bed-side monitors, infusion pumps, and a call light system. Table 3 shows that each nurse received 30 alarms/alerts (only three of which were important and actionable) plus 5 call-light system alerts for each of the two 90-minute segments that both included distribution of patient medications. In the simulation, five different types of patients were replicated in four sets. The patient with methicillin-resistant Staphylococcus Aureus (MRSA) required that nurses don personal protective equipment (PPE) including gowns, gloves, and face shields. Pre-planned task assignments for nurses were designed to require heavy workload and multitasking for approximately the first 60 of each 90-minute segment.

**Table 3:** Patient simulation scenarios with actionable and non-actionable alarms (n depicts the number of events).

Patient Diagnosis and Scenario Description	Clinical Risks Replicated in Simulation	Scenario Part-A: 7:30 AM—9:00 AM simulation time			Scenario Part-B: 4:30 PM—6:00 PM simulation time		
		Adverse Event	Actionable Alarms	Non-Actionable Alarms	Adverse Event	Actionable Alarms	Non-Actionable Alarms
Status/post small bowel resection and methicillin-resistant *Staphylococcus Aureus*	Pain management and associated adverse events of analgesia, sepsis, fluid and electrolyte imbalances, infection control	None	None	IV pump: ‘Air in Line’, 0.9% NaCl infusion (n = 1)	Early sepsis	Low systolic blood pressure (with non-alarming rise in heart rate)	IV pump: ‘Low Battery’ (n = 1)
Heart failure exacerbation with episodes of hypotension	Fluid overload, hypoxemia, acute renal failure, cardiogenic shock	None	None	SpO_2_ monitor: ‘No Signal’ (n = 6); Low systolic blood pressure (n = 7)	None	None	SpO_2_ monitor: ‘No Signal’ (n = 6); Low systolic blood pressure (n = 7)
Status/post radical prostatectomy with urinary catheter and PCA with morphine infusion	Pain management and associated adverse events of analgesia, infection, postsurgical hemorrhaging	Respiratory depression	Low SpO_2_ and low respiratory rate	IV pump: ‘Low Battery’ (n = 1)	Occluded PCA line	PCA: ‘Occlusion’	None
Deep vein thrombosis in lower extremity on heparin infusion	Failure to achieve therapeutic anticoagulation, pulmonary embolism	Occlusion in IV line with heparin infusion	IV pump: ‘Occlusion’	SpO_2_ monitor: ‘No Signal’ (n = 3)	Suspected pulmonary embolism	Low SpO_2_ (with non-alarming rise in respiratory and heart rate)	IV pump: ‘Air in Line’, 0.9% NaCl infusion (n = 1); SpO_2_ monitor: ‘No Signal’ (n = 3)
Chronic bronchitis and acute pneumonia with episodes of hypoxemia and receiving IV antibiotic therapy	Severe hypoxemia/ hypercapnia, antibiotic resistance, sepsis	Unanticipated onset of bradycardia	Low heart rate with non-alarming drop in blood pressure	IV pump: ‘Infusion Complete’, 0.9% NaCl (n = 1); SpO_2_ monitor: Low SpO_2_ (n = 9)	none	none	IV pump: ‘Infusion Complete—Vancomycin’ (n = 1); SpO_2_ monitor: Low SpO_2_ (n = 9)

IV = intravenous; SpO_2_ = hemoglobin oxygen saturation as measured by pulse oximetry; PCA = patient controlled analgesia

Both scenario segments included a set of emergent changes from the normal simulation baselines for each patient (see Table 3). Frequencies of alarms/alerts matched rates from literature for acute care settings– 96 alarms per bed per day [59]. A set of 5 patients will therefore have about 30 alarms/alerts per 90 minutes– 90% of which are not actionable or important. A scenario server introduced these deviations that caused alarm triggers on the simulated monitors and intravenous infusion pumps. The vital signs for every patient were individually generated using a custom autoregressive–moving-average time series algorithm. It was parameterized using samples of archived real patient data and a model of the five different types of patients from the scenario.

In the two scenario segments, each set of five patients was designed to experience three clinically-important and actionable alarm/alert events, as well as 27 non-actionable alarms/alerts. In addition, each patient had one call-light alert event per segment. Therefore, each nurse received a total of 35 alarms/alerts through the HAIL-CAT smartwatch during each of the two 90-minute scenario segments. A bedside physiological monitor, two intravenous infusion pumps (including patient controlled analgesia for one patient) were simulated in every room with an Android tablet created for the experiment. Simulated monitors showed vital signs for every patient updated once per second, including: heart rate, systolic/diastolic blood pressure, respiratory rate, and SpO2 (hemoglobin oxygen saturation as measured by pulse oximetry). Alarm thresholds were configured as follows: SpO2 < 90%, heart rate < 50 and > 120 beats per minute, respiratory rate < 10 and > 30 breaths per minute, systolic blood pressure < 90 and > 160 mm Hg, diastolic blood pressure < 50 and > 90 mmHg. Conducting the experiment required 18 experimental staff: four observers (one per nurse participant); four ‘family member’ confederates (one per nurse participant); two nursing assistants; two nurse practitioners; one charge nurse; four technical support); and one phone responder in a separate room answering all calls to patients’ physicians and other hospital departments.

Every aspect of the design and development of the simulation scenario prioritized creating the highest possible clinical realism representing a typical U.S. hospital acute care unit. Configuration of each part of the scenario leveraged clinical expertise and data from actual hospitals (medical literature and raw de-identified samples) [94]. The scenario was crafted to present the most common: kinds of patients; patient vital signs; device alarm threshold settings; frequency of alarms/alerts; ratio of important alarms/alerts to non-actionable alarms/alerts; nurse tasking; medications; procedures; care coordination; etc. Many features of the scenario were leveraged from other pre-existing clinical patient simulation scenarios (unrelated to this experiment) that had been iteratively developed and tested for training nurses in highly realistic situations. Leveraged pieces included: clinical workflows; clinical tasking; medication scheduling; care coordination; and assessment. Also, the experimental staff of clinical actors were experienced in running high-fidelity patient simulation for other projects, and were instructed to maximize the realism of the experience for nurse subjects. Three clinical expert nurse researchers with no tie or understanding of the experimental hypothesis contributed to and reviewed the scenario. In exit interviews, nurse participants were asked to comment on the degree to which they felt the scenario was realistic. There was consensus that the scenario felt extremely real.

### Wearable attention aid prototype

**Fig 1:**
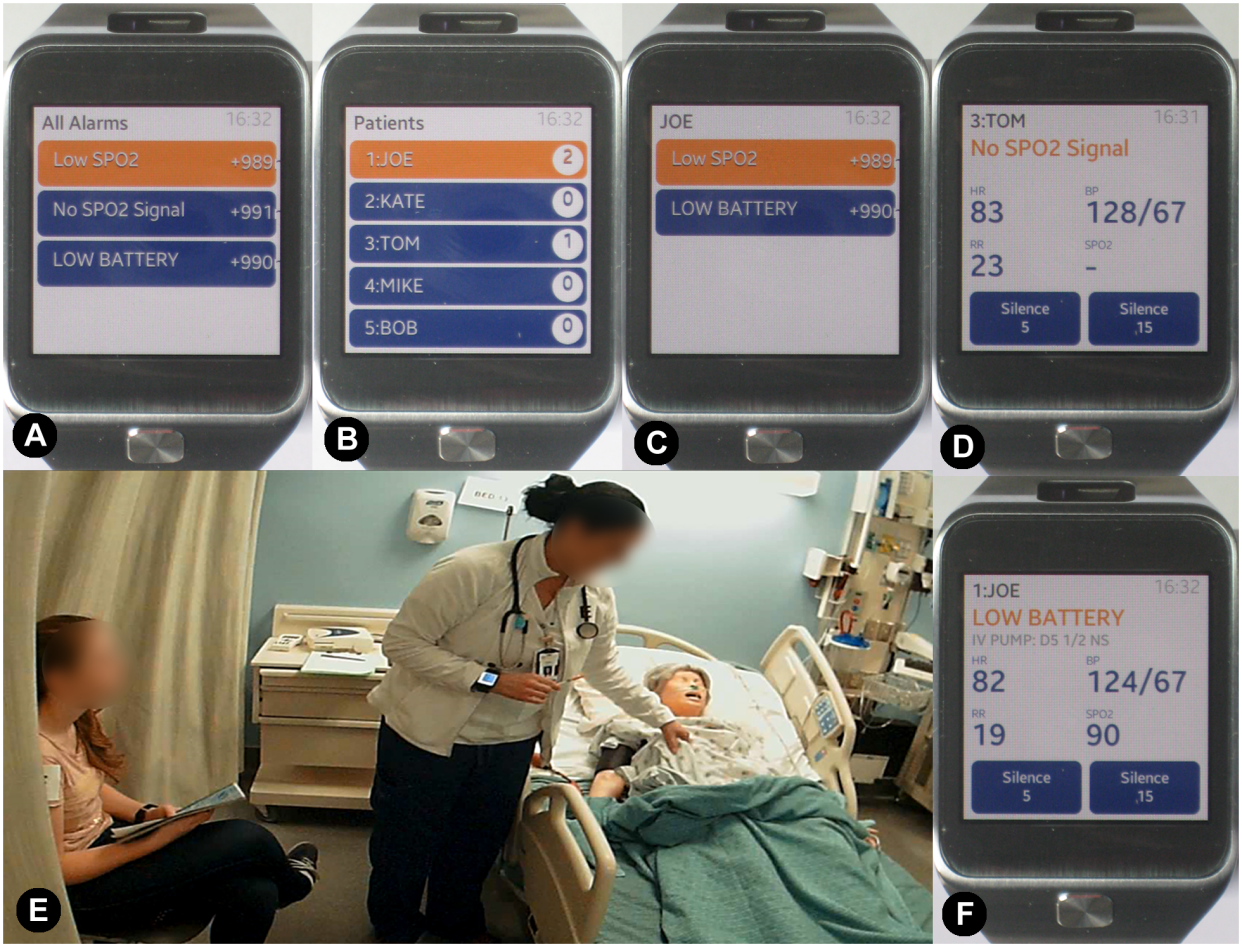
The HAIL-CAT (Human Alerting and Interruption Logistics—Clinical Alarm Triage) wearable attention aid prototype. The smartwatch application has four screens: (A) list of all alarms/alerts (blue marks silenced; orange marks not-silenced); (B) home screen list of five patients (including number of current alarms/alerts); (C) list of alarms/alerts for selected patient; (D & F) alarm/alert announcement with message and vitals context. "E" shows a nurse participant (standing and wearing the prototype on her right wrist). She is checking a "patient" (a patient simulation mannequin in the bed) while speaking with a "family member" (experimental confederate) who sits nearby. In addition to triaging alarms/alerts, the smartwatch enabled nurses to check the vital signs for any patient at any time by selecting the patient from the home screen. The vital signs screen is the same as "D" or "F," but without the alarm/alert message and "silence" buttons.

The HAIL-CAT wearable prototype provides a set of context-enabled alarm notification services to support users’ metacognition for interruption triage (see Fig 1). It was implemented on Samsung Gear 2 smartwatches (Samsung Corp., Seoul, South Korea) using Java software technologies (Oracle Corp., Redwood City, CA, USA) and commercial-off-the-shelf (COTS) Wi-Fi networking (a standard wireless local area network technology) [94]. The smartwatch function was supported by a server-side integrated data environment and analytics engine. A central experimental simulation server provided: patient vitals every second; scenario deviations; data integration; alarm generation; attention-aiding alarm mediation and delivery services; and user-directed information access services.

During the experiment, nurses each attended to five simulated patients. When an alarm occurred, the responsible nurse received an announcement via a short, non-obtrusive vibration of the smartwatch on her/his wrist. The nurses then could look hands-free to see: which patient was experiencing an event; what room they were in; the details of the alarm/alert; and the context of associated patient vital signs (heart rate, blood pressure, respiratory rate, and SpO2). The nurse was also presented with the option to either silence the alarm for a short period of time (5 or 15 minutes) with the press of a button, or to do nothing and allow the bedside alarm audio to continue.

The user interface (UI) for the HAIL-CAT smartwatch presented nurses with two types of interactive services relative to her/his set of five patients. First, it provided an alert notification service for delivery of integrated alarm/alert messages from bedside monitors, infusion pumps, and call light system events. Second, HAIL-CAT supported at-will checking on the status of every patient. Contextual information about patients’ physiological status (heart rate, blood pressure, respiratory rate, and SpO2) was included in every alarm announcement. This enabled nurses to dynamically manage the meta-level work of organizing their multitasking schedule amid frequent changes to patients’ status. With the smartwatch they were able to both maintain awareness of important unexpected changes to patients’ status or risks to care delivery, as well as perform pre-planned care tasking. Balancing these two objectives is a common nurse challenge and can be referred to as ’track-while-scan’ [94]. ’Track’ work tracks the performance of pre-planned care, and ’scan’ work continually scans for emergent of new unexpected problems.

### Technical implementation and generalizability

The full experimental system includes not only the HAIL-CAT wearable, but also all the machinery for driving the events and functions of the entire 20-bed acute care unit simulation facility. Three key requirements for design of the experimental system were: (1) to serve as a research facilitation for the quantitative repeated measures experiment reported here; (2) deliver the HAIL-CAT functionality within this experiment, and (3) to only include technology functions that have been proven feasible within real hospital environments. Relative to these requirements, the design of the experimental system was free to include technical simplifications or mock-ups as conveniences for whatever had already been proven technically feasible elsewhere.

The design of the experimental platform assumes a hospital acute care unit (in simulation) environment with multiple technologies already in place, including: physiological monitors and IV pumps in every patient room, and a local area network (LAN) with Wi-Fi. It also assumes the prior installation of a sophisticated middleware system that integrates data across a LAN from these monitors, IV pumps, and a call light system. With this infrastructure in place, the introduction the HAIL-CAT platform smartwatches paired with cellphones is technically feasible. For the experiment, the actual 20-bed patient simulation facility used for this study did not already have all these components installed. Instead of acquiring and deploying the missing infrastructure, the environment was prepared for the experiment by introducing mock-ups that provided sufficient realism to cover the experimental scenario.

The implementation of this experimental platform is described in another technology-oriented publication (for details see [94]). In summary, system implementation is centered on a single Linux OS server running on a laptop PC and connected by Ethernet to a Linksys N600 dual band Wi-Fi router. This server establishes a Wi-Fi LAN for controlling: 20 Asus Nexus 7 Android tablets (one in each of the 20 patient rooms); and four pairs of Samsung Galaxy S5 Android cellphones and the Samsung Gear 2 smartwatches (one phone/watch pair for each of the four nurse participants). Custom Java-based software running on the Linux server, simulates vital signs for all 20 patients. It delivers wireless two-way communication (Wireless 802.11 TCP connections) through a common message bus and routes relevant patient vital signs once per second to every tablet and cellphone. The cellphones then communicate this to the smartwatches through Bluetooth Low Energy (BLE). The cellphones ran custom Java-based software to drive interactive UIs on both the cellphone and the smartwatch. The smartwatch ran a custom web app on its web browser that communicated with the paired cellphone. The tablets ran custom Java-based software to communicate with the Linux server, and to deliver interactive UIs that simulate a physiological monitor and an IV pump in each patient room. Mock integration with the call system, the SimMan-2G patient mannequins, and the Omnicell automated medication and supply cabinets was done manually by experimental confederates/researchers and automated. The architecture design concentrates processing on the Linux server to simplify device-based software.

The successful function of this system for the experimental provides evidence of the technical feasibility for conducting quantitative repeated measures experiments at full hospital unit scale. This includes, supporting high fidelity workflows of entire teams of clinical participants delivering simultaneously care for a large number of simulated patients (sophisticated patient mannequin), and integration with novel device prototypes (the HAIL-CAT wearable). This strong technical result highlights the potential for a new qualitative experimental research approach option for improving a wide variety of healthcare issues.

With one exception, the experimental platform also provides evidence of the technical feasibility of the HAIL-CAT wearable for deployment in a real hospital. Implementation would only require integration with a middleware system and an inexpensive hands-free wearable platform with a new custom application—potentially based on existing COTS. The one exception is the single function that allows nurses to temporarily silence bedside alarms remotely from their smartwatches. To enable nurses to silence bedside alarms from a wearable would require remote integration with the controls of medical devices—in this case physiological monitors and IV pumps. The technology to enable this is low risk, however there are complex issues regarding regulation and vendor support. Although nurses commented positively in qualitative interview about the utility of remotely silencing alarms, removal of this one feature would not change the observed quantitative results on the primary metric—improved time to respond.

### Experimental data collection procedures

Upon arrival, each team of four nurses read and signed a consent form. They received an orientation to the simulation lab, mannequins, study procedures, clinical responsibilities, and roles of the confederates and observers. Nurses then reviewed a written change of shift report with information on their five assigned patients. This included: patient histories, summary of recent changes or events, and recent vital signs and laboratory results. Nurses then received a task checklist for each patient for the first 90-minute scenario segment. This included: acquiring vital signs, conducting a physical assessment, administrating scheduled and PRN (as needed) medications, and communications with other healthcare team members.

The condition order (HAIL-CAT vs. no smartwatch) was assigned for each group arbitrarily according to a balanced schedule. Before using HAIL-CAT, participants received 10 minutes of training on the technical operation and function of the wearable attention aid prototype. To avoid biasing the results, training did not include any information about strategies for using the wearable attention aid in practice or reveal the experimental hypothesis or metrics. Nurse participants were asked to focus on their role of performing the clinical simulation scenario. Participants were instructed that they had the freedom to use the wearable prototype (or not) however seemed best to them relative to this role. During the experiment, four observers (with a hat-mounted high-definition video cameras) shadowed nurse participants (one observer for each nurse) and recorded times for all patient visits. Following completion of the two 90-minute scenario segments nurses completed an exit questionnaire and participated in a semi-structured interview with their observer.

### Data analysis

Statistical analyses were performed using MathWorks MATLAB version R2014b. Assumptions required for parametric statistical methods were analyzed. Table 4 shows the results of test of normality on data for the primary metric—time to response to important alarms/alerts. The Anderson-Darling test [127] and the Jarque-Bera test show that the data are not normally distributed; nonparametric statistical methods are indicated. P-values in Table 4 are probabilities that the data are normally distributed, so small p-values mean that it is very unlikely that the data represent samples from a normal distribution. Note, non-normality of data distribution does not have any meaning relative to the quality of the experiment or data. Knowing this about the data is only useful for determining which class of statistical methods is more appropriate for analyzing observations [128].

**Table 4:** Tests of normality on data for time to respond to important alarms.

Statistical Test	Output	Control: Without Aid	Treatment: With HAIL-CAT Aid
Anderson-Darling test	p-value	p = 0.001	p = 0.001
	AD Statistic	2.202	3.626
	Critical Value	0.739	0.739
Jarque-Bera test	p-value	p = 0.001	p = 0.001
	JB Statistic	62.780	77.509
	Critical Value	4.912	4.932

Because the data are not normally distributed, nonparametric statistical options were chosen for: hypothesis testing, and analysis for possible side-effects. For hypothesis testing, the experiment produced interval data with two-sample related or matched samples. For this kind of data, Siegel [129] suggests the Permutation test for paired replicates, or the Wilcoxon signed ranks test. Both are computed for the hypothesis test. Descriptive statistics include the sum, mean, standard deviation (SD), median (mdn), and median absolute deviation (MAD; a measure of statistical dispersion similar to standard deviation). Analyses for secondary effects and group effects were performed with the Mann-Whitney U-test (also called the Wilcoxon rank sum test); and Brown–Forsythe test to analyze the significance of differences in dispersion or variation (not values) between two samples. Alpha was set at 0.05 for all analyses.

## Results

Analysis of data from empirical observation show that introduction of the HAIL-CAT wearable attention aid enabled nurses to quickly triage unfiltered alarms/alerts and respond more quickly to important problems with patients’ status and care delivery. With the aid, the median improvement for individual nurses was 118% compared to their performance without the wearable. Simulated patients across the unit received 148% faster nurse response overall after the onset of an important alarm/alert. This evidence highlights the possibility of improving patient safety by introducing metacognitive aids to better use existing alarms/alerts (approach ’H’ from Table 1).

### Primary metric: Time to respond to important alarms

Table 5 shows the data and descriptive statistics for the primary metric. In both of the two scenario parts of the experiment, each nurse received 30 alarms (plus five call-light alerts), with only three alarms being important and clinically actionable. The time data in Table 5 (control and treatment columns) is the time to respond for only the three important alarms for each nurse split by condition. The response times for the other 27+5 non-actionable alarms/alerts are not included here. The columns of mean values from Table 5 were used to test the hypothesis. Note, Table 5 does not show the condition order. Balanced randomization of treatment order for the repeated measures provides sufficient control to neutralize possible experimental confounds related to condition order, learning, or fatigue.

**Table 5:** Time to respond to important alarms (minutes).

Subject		Control: Without Aid						Treatment: With HAIL-CAT						Difference		
ID	Group	Scenario Part	Response Time to Three Important Alarms (min.)			Sum (min.)	Mean (min.)	Scenario Part	Response Time to Three Important Alarms (min.)			Sum (min.)	Mean (min.)	Diff. of Sums (min.)	Diff. of Means (min.)	RPD of Means
**1**	1	A	1.02	10.67	20.97	32.65	10.88	B	0.52	0.32	0.03	0.87	0.29	-31.78	-10.59	3,667
**2**	1	A	0.20	9.88	0.33	10.42	3.47	B	11.13	6.32	0.17	17.62	5.87	7.20	2.40	-41
**3**	1	A	0.48	28.10	31.90	60.48	20.16	B	1.85	12.85	11.85	26.55	8.85	-33.93	-11.31	128
**4**	1	A	26.45	1.23		27.68	13.84	B	0.20	8.63	8.58	17.42	5.81	-10.27	-8.04	138
**5**	2	B	3.72	0.07	8.07	11.85	3.95	A	0.50	0.33	6.88	7.72	2.57	-4.13	-1.38	54
**6**	2	B	14.42	10.02	0.03	24.47	8.16	A	2.27	7.05	26.37	35.68	11.89	11.22	3.74	-31
**7**	2	B	1.57	0.07	5.60	7.23	2.41	A	3.42	1.13	5.82	10.37	3.46	3.13	1.04	-30
**8**	2	B	0.08	8.12	12.08	20.28	6.76	A	3.37	3.93	3.57	10.87	3.62	-9.42	-3.14	87
**9**	3	B	24.98	0.03	11.35	36.37	12.12	A	2.72	3.57	3.58	9.87	3.29	-26.50	-8.83	269
**10**	3	B	11.88	2.63	0.18	14.70	4.90	A	0.42	4.17	25.40	29.98	9.99	15.28	5.09	-51
**11**	3	B	3.83	5.57	7.12	16.52	5.51	A	0.52	3.17	3.05	6.73	2.24	-9.78	-3.26	145
**12**	3	B	0.03	11.72	18.65	30.40	10.13	A	6.52	6.62	1.47	14.60	4.87	-15.80	-5.27	108
**13**	4	A	1.18	8.60	15.10	24.88	8.29	B	3.17	0.17	0.53	3.87	1.29	-21.02	-7.01	544
**14**	4	A	4.28	9.67	25.63	39.58	13.19	B	10.40	3.12	0.47	13.98	4.66	-25.60	-8.53	183
**15**	4	A	25.13	30.92	63.17	119.22	39.74	B	0.65	0.03	0.67	1.35	0.45	-117.87	-39.29	8,731
**16**	4	A	3.52	29.10	19.43	52.05	17.35	B	3.62	5.02	18.42	27.05	9.02	-25.00	-8.33	92
**Sum**			122.78	166.38	239.62	528.78	180.88		51.25	66.42	116.85	234.52	78.17	-294.27	-102.70	13,992
**Mean**			7.67	10.40	15.97	33.05	11.30		3.20	4.15	7.30	14.66	4.89	-18.39	-6.42	875
**SD**			9.75	10.28	16.15	27.29	9.11		3.40	3.55	8.79	10.45	3.48	-16.83	10.18	2,279
**Mdn**			3.62	9.13	12.08	26.28	9.21		2.49	3.75	3.58	12.43	4.14	-13.86	-6.14	118
**MAD**			3.28	5.03	8.12	10.83	4.15		1.91	2.74	3.21	5.44	1.81	-5.39	3.73	149

The ’Difference’ columns in Table 5 show the difference in response time between the control and treatment conditions. The relative percent difference (RPD) score is the ratio of the mean difference in response time between the two conditions divided by the mean time to respond while using the HAIL-CAT wearable. Alternatively stated, it is the size of the effect of introducing HAIL-CAT relative to the end result of using it. Positive RPD scores indicate improved response times (relative shorter response times) when using the HAIL-CAT wearable compared to the control condition. Negative RPD scores mean that using HAIL-CAT resulted in longer times. An RPD score of 0% would mean no change between the two conditions.

For example, the average response time for important alarms/alerts for RN number 8 (see Table 5) was 6.76 minutes without the smartwatch, and 3.62 minutes with the smartwatch. For this RN, introduction of the smartwatch caused her/him to respond 3.14 minutes faster on average to important alarms/alerts. The RPD score for this participant is (3.14 / 3.62) * 100 = 87%. This indicates that the RN’s mean response time to important alarms while using the HAIL-CAT wearable was 87% faster than her/his mean response time in the control condition—an 87% improvement. The amount of time reduced by introduction of HAIL-CAT for RN-8 (3.14 min. relative to the control condition) is similar to RN-8’s response time using HAIL-CAT (3.62 min.).

**Fig 2:**
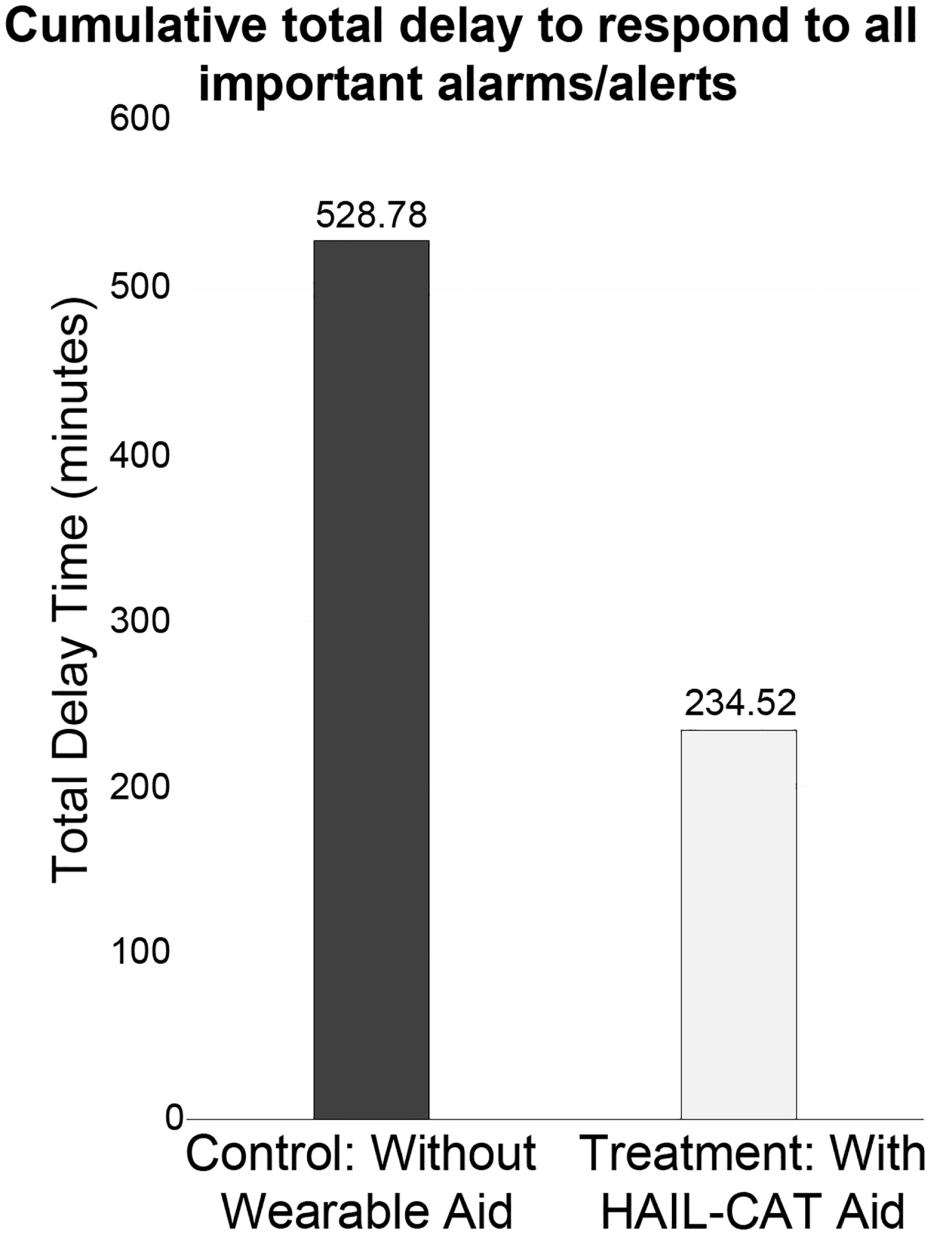
Cumulative total time for all 16 nurses to respond to important alarms (minutes). These are sums of response times (split by condition) for all experimental trials across the entire simulated acute care unit.

**Fig 3:**
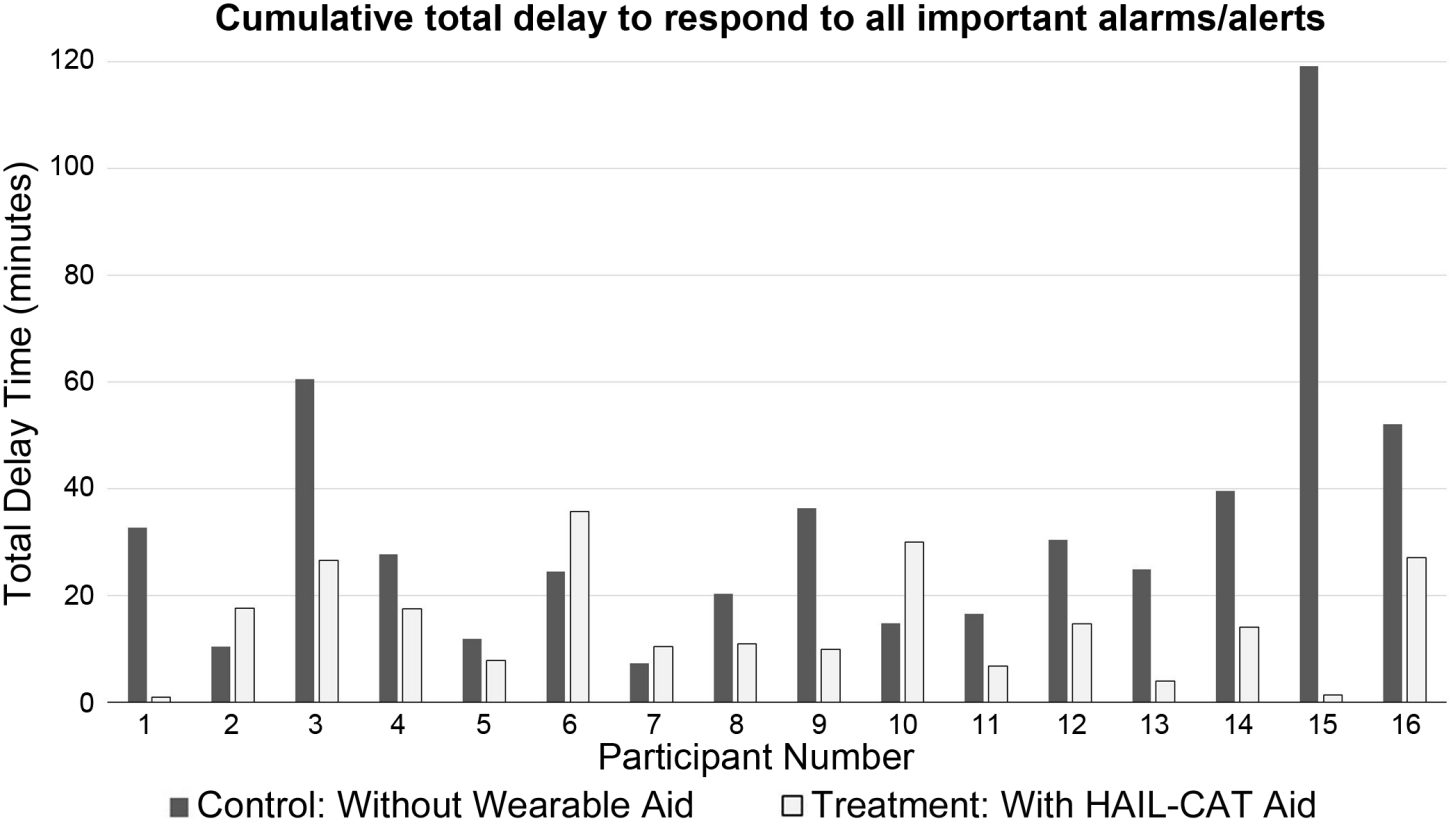
Cumulative total time to respond to important alarms split by participant (minutes). These are sums of response times (split by condition and participant) for all experimental trials across the entire simulated acute care unit.

Fig 2 shows the cumulative total time delay for all important alarms split by experimental condition. The graph shows the totals for 48 important alarms for the control condition, and 48 important alarms for the treatment condition (three important alarms delivered to each of 16 participants for each of the two experimental conditions). Fig 3 shows the same data split by participant.

Ranking participants by their individual RPD scores shows different types of effects across the four quartile subsets. For the top quartile of participants ranked by RPD score, the introduction of the HAIL-CAT wearable aid caused an extreme and dramatic positive effect on responding to important alarms (8,731%, 3,667%, 544%, and 269% improvement). While wearing HAIL-CAT attention aid, these nurses were observed to consistently use the wearable to triage new alarms/alerts and respond quickly to the patients’ bedside at the beginning of important changes. With additional training and/or experience using the HAIL-CAT smartwatch, a larger proportion of nurses may be able to achieve this extremely high level of performance and reliability.

Participants in both the second and third quartiles (the middle half of all participants) all received substantial benefit from using HAIL-CAT (183%, 145%, 138%, 128%, 108%, 92%, 87%, and 54%). This large positive effect seems to be linearly proportional to their performance on the control condition.

The bottom quartile of participants (by RPD score rank) was negatively affected by the introduction of HAIL-CAT (-30%, -31%, -41%, and -51%). Interestingly, this quartile included three of the top four performers on the control condition (without the wearable attention aid), who had displayed advanced skills in prioritization and multitasking. Their advanced strategies, however, seemed disrupted by the introduction of the smartwatch and participants did not have sufficient time in the experimental scenario to adapt.

A curious pattern emerges by sorting participants by their RPD performance scores (time to respond to important alarms/alerts) in the control condition—when not using the wearable. Of the top eight performers on the baseline condition, half (four) had some benefit with the aid, and half (four) had some negative effects. Of the bottom eight performers on the baseline condition, all benefited from using the wearable. It is unclear whether this is a meaningful pattern, and is something that merits additional investigation.

### Hypothesis test

Every nurse did one part of the two-part scenario while wearing the smartwatch and the other part of the scenario without the smartwatch. The "Difference" column from Table 5, shows the relative change in time to respond to important alarms for each nurse comparing their times while wearing the attention aid to not wearing it. The hypothesis tests calculate the two-tailed likelihood that these differences could occur by chance. Table 6 summarizes the results of hypothesis tests of the within-subjects single factor experimental design using Siegel’s Permutation Test [129] and the Wilcoxon Signed Ranks Test.

**Table 6:** Relative within-subjects response to important alarms/alerts.

	Median Difference (minutes)	Relative Percent Difference	Siegel’s Permutation Test		Wilcoxon Signed Ranks Test	
			W Statistic	p-value	W Statistic	p-value
Within-subjects comparison of response time to important alarms/alerts with and without the wearable attention aid	-6.14 minutes	118%	183	p = 0.006	119	p = 0.008

Evidence confirms the hypothesis that introduction of the HAIL-CAT wearable attention aid significantly improved nurse response time to important alarms/alerts. The median improvement for each nurse to responded to important alarms/alerts was about 6.14 minutes faster when she/he was wearing the smartwatch compared to when she/he was not (Tables 5 and 6). This statistically significant finding suggests the potential of this approach to intervene earlier in the onset of adverse events.

This study shows the potential to dramatically improve nurse response time at the bedside after the onset of alarms/alerts associated with important actionable events. The HAIL-CAT wearable aid empowered most nurses to more easily use existing alarm alert signals, without reducing alarm frequency or improving alarm quality. This finding does not negate the potential for additional improvement through improved alarm generation. However, it does highlight the high potential of an approach that was previously unrecognized for healthcare.

### Analysis of potential secondary effects by trial condition

Beyond the successful improvement of the primary metric, introduction of the wearable attention aid would not be practical if it caused negative side-effects to other aspects of nurses’ work. So, in addition to the favorable hypothesis test, a set of other nurse performance metrics are analyzed for possible effects of the introduction of the wearable attention aid. Hospital unit-level nursing performance for the experiment can be split into two conditions depending on whether the four nurses were wearing the HAIL-CAT smartwatch prototype or not. In this split of the data (referred to here as "trial condition"), any unintended differences due to scenario effects from the different parts of the two-part scenario are nullified by the balanced randomization of treatment orders. Were there any unit-level differences in nurse performance overall between when they were using the smartwatch or not?

Beyond the successful improvement of the primary metric, introduction of the wearable attention aid would not be practical if it caused negative side-effects to other aspects of nurses’ work. In addition to the favorable hypothesis test, a set of other nurse performance metrics are analyzed for possible effects of the introduction of the wearable attention aid.

There are abundant articles documenting possible negative side-effects of interrupting people while they are working [29–32,49,130–134]. These results highlight the caution that must be taken in introducing new alarms/alerts into a work setting. Other research shows that the scope and impact of these side-effects is highly variable, and potentially can be mitigated in practice with innovative human-computer interaction (HCI) designs that support users’ metacognitive processes [69,100,135,136]. Prior work with strong internal validity has shown the benefits of negotiation-based metacognitive services for mitigating the negative side-effects of interruption [100,102].

This present study focuses on using these results to enable users to extract whatever utility from the alarms/alerts that are actually present in practice (see approach ’H’ from Table 1). The scenario used for this study, therefore, did not vary the number or quality of alarms/alerts that nurse participants had to deal with between the two experimental conditions. Nurses were exposed to equivalent numbers of alarm/alert-based interruptions to their primary work regardless of whether they were working the control or treatment conditions. Any difference in performance of primary metrics, or of secondary side-effects, were caused only by differences in how alarm/alert-based interruptions were delivered.

The experimental design chosen for this study emphasizes a balance between experimental control and clinical realism to maximize the combination of both internal and external validity. As a result, many of the interesting subtle effects of interruption on user cognition and metacognition were not measured because the available methods for taking these measurements would themselves have been prohibitively disruptive. Some interesting types of potential metrics were, therefore, not taken. The potential reduction to external validity was too great. Instead, nurse participants were allowed to perform their mobile clinical duties in a highly realistic way. This included representative high volumes of clinically-realistic interruptions, without adding more distraction from experimenter intervention/interference.

The two trial conditions ("with" and "without") are compared to analyze the potential effects on a set of other nursing metrics. Table 7 lists nine metrics ’A’ through ’I’. Note that the primary metric is included in this list, because it is useful to also analyze its trial condition for any potential side-effects not revealed in the within-subjects analysis. Table 8 shows the results of analyses across these metrics.

**Table 7:** Trial condition metrics for analysis of potential secondary effects.

Metric	Description
(A) Response to actionable alarms	Time for nurse to respond at the bedside from onset of important actionable alarms (in minutes).
(B) Response to Non-Actionable Alarms	Time for nurse response at the bedside from onset of non-actionable or unimportant alarms (in minutes).
(C) Time with Patients	Total time spent in the room with patients per nurse out of a total of 90 minutes for each of two scenario parts (in minutes).
(D) Number of visits with Patients	Total number of individual patient visits per nurse (count).
(E) Environmental Awareness	Percent of environmental patient safety issues scripted into the scenario that were noticed and fixed by nurses.
(F) Response to Patient Requests	Percent of patient requests scripted into the scenario that were accomplished by nurses.
(G) Walking	Total footsteps per nurse as measured by pedometer (count).
(H) Consults	Total consultations (per four-nurse team) with clinical authority about an important change in a patient’s status or care.
(I) Alarms Sounding (Noise)	Total number of simultaneously sounding alarms for each 90-minute scenario part (count)

**Table 8:** Analyses of potential secondary effects by trial condition.

		(A) Response to actionable alarms	(B) Response to Non-Actionable Alarms	(C) Time with Patients	(D) Number of visits with Patients	(E) Environmental Awareness	(F) Response to Patient Requests	(G) Walking	(H) Consults	(I) Alarms Sounding (Noise)
	Units	minutes	minutes	minutes	count	percent	percent	steps	count	count
Control: Without Aid	Median	8.12	3.62	38.12	29	20	100	1105	8	11
	MAD	6.98	3.28	14.88	10.5	20	0	170	4	5.814
	Min	0.03	0.02	19.23	14	0	0	743	3	1
	Max	63.17	49.73	77.67	54	80	100	1980	13	26
	Sum	528.78	3725.20	682.20	485	n/a	n/a	17544	32	66161
Treatment: With HAIL-CAT Aid	Median	3.27	1.58	41.73	31	20	100	1297	9	3
	MAD	2.76	1.31	4.33	5	0	0	246	0.5	1.929
	Min	0.03	0.03	25.90	15	0	0	675	8	0
	Max	26.37	45.42	65.05	45	100	100	2557	10	8
	Sum	234.57	2049.83	662.87	496	n/a	n/a	18482	36	n/a
Effect on value: Mann-Whitney U	RPD	148	129	-9	-6	0	0	-15	-11	267
	p-value	0.016	< 0.001	0.925	0.597	0.504	0.676	0.182	1.000	< 0.001
	U	2.401	6.529	-0.094	-0.528	0.669	0.418	-1.336	18.000	77.148
Effect on variance: Brown-Forsythe	RPD	153	150	244	110			-31	700	201
	p-value	0.001	< 0.001	0.004	0.084	0.567	0.748	0.336	0.002	< 0.001
	F	11.670	37.708	10.014	3.195	0.336	0.105	0.962	29.400	5769.080

MAD = median absolute deviation

A significant improvement in "non-actionable alarm checking" showed that nurses also responded faster on average to non-actionable alarms/alerts when wearing the smartwatch. The greatly reduced cognitive effort for checking each alarm signals enables nurses to fit in times to visit patients sooner even when the change is not clinically important. Results also show that this gain in performance did not cause harmful side-effects to nurses’ performance on pre-planned tasks. Note, the differences in scenario scheduling of important alarms does not support any conclusion about the observed overall faster response time for non-actionable alarms compared with response time for important alarms. These secondary measures show no significant change in either value or dispersion of scores, with two exceptions. Use of the wearable attention aid caused a significant positive reduction in variation of total time spent with patients and for number of consults with the nurse practitioners. Table 9 summarizes these conclusions.

**Table 9:** Conclusions of analyses results for potential secondary effects by trial condition.

Metric	Expected Effect	Observed Effect	Negative Side-Effects	Conclusion
(A) Response to actionable alarms	Yes, faster is better	Yes, very positive	None	Two and a half times faster response may result in early intervention, thus potentially protecting patients from adverse events, and with significantly less variation in response time.
(B) Response to Non-Actionable Alarms	No	Yes, very positive	None	On average, nurses were checking non-actionable alarms significantly faster and with significantly less variation.
(C) Time with Patients	No	Not on time spent, but yes on consistency	None	No difference in the time nurses spent with patients, however there was significantly less variation among nurses (an improvement).
(D) Number of visits with Patients	No	No	None	No difference in the number of patient visits per nurse with a decrease in variability that approaches significance.
(E) Environmental Awareness	No	No	None	No significant effect (positive or negative) on noticing and fixing environmental patient safety issues.
(F) Response to Patient Requests	No	No	None	No significant effect (positive or negative) on accomplishing patients’ requests.
(G) Walking	No	No	None	No significant effect (positive or negative) on distance nurses walk.
(H) Consults	No	Not on count, but yes on consistency	None	No significant effect in the frequency of consultations, but sizeable improvement in consistency in the frequency of consultations.
(I) Alarms Sounding (Noise)	No	Yes, very positive	None	The unit was more than three times quieter and with significantly less variation in noise levels.

### Analysis of potential secondary effects within subjects

In addition to possible secondary effects by trial condition, an analysis for potential secondary effects within subject is also useful. Table 10 describes this analysis. Unlike the results of Tables 8 and 9 that are split by trial condition, these results describe whether introduction of the wearable attention aid caused relative individual change in performance for a significant proportion of nurses. Results show relative change across individual nurses for how much the introduction of the smartwatch affected behaviors. The primary metric (’A’) is omitted because this analysis is the same as the Wilcoxon signed ranks test used in the hypothesis test. Results confirm that introduction of the smartwatch caused no negative side-effects on nurse performance across any of these metrics. This is further confirmation that introduction of the smartwatch prototype, not only caused a positive impact on the primary metric, but it did not negatively affect nurse performance in other secondary ways.

**Table 10:** Analyses of potential secondary effects within subjects.

	Relative Median Change with Smartwatch	Percent Change	p-value (Wilcoxon signed ranks)	W Statistic	Negative Side-Effects
(B) Response to Non-Actionable Alarms	-2.16 minutes	160%	p = 0.005	123	None
(C) Time with Patients	3.42 minutes	14%	p = 0.796	63	None
(D) Number of visits with Patients	-0.50 visits	-2%	p = 0.858	56.5	None
(E) Environmental Awareness	0%	0%	p = 0.753	31	None
(F) Response to Patient Requests	0%	0%	p = 0.844	12	None
(G) Walking	207 steps	-12%	p = 0.339	26	None

### Subjective outcomes

**Fig 4:**
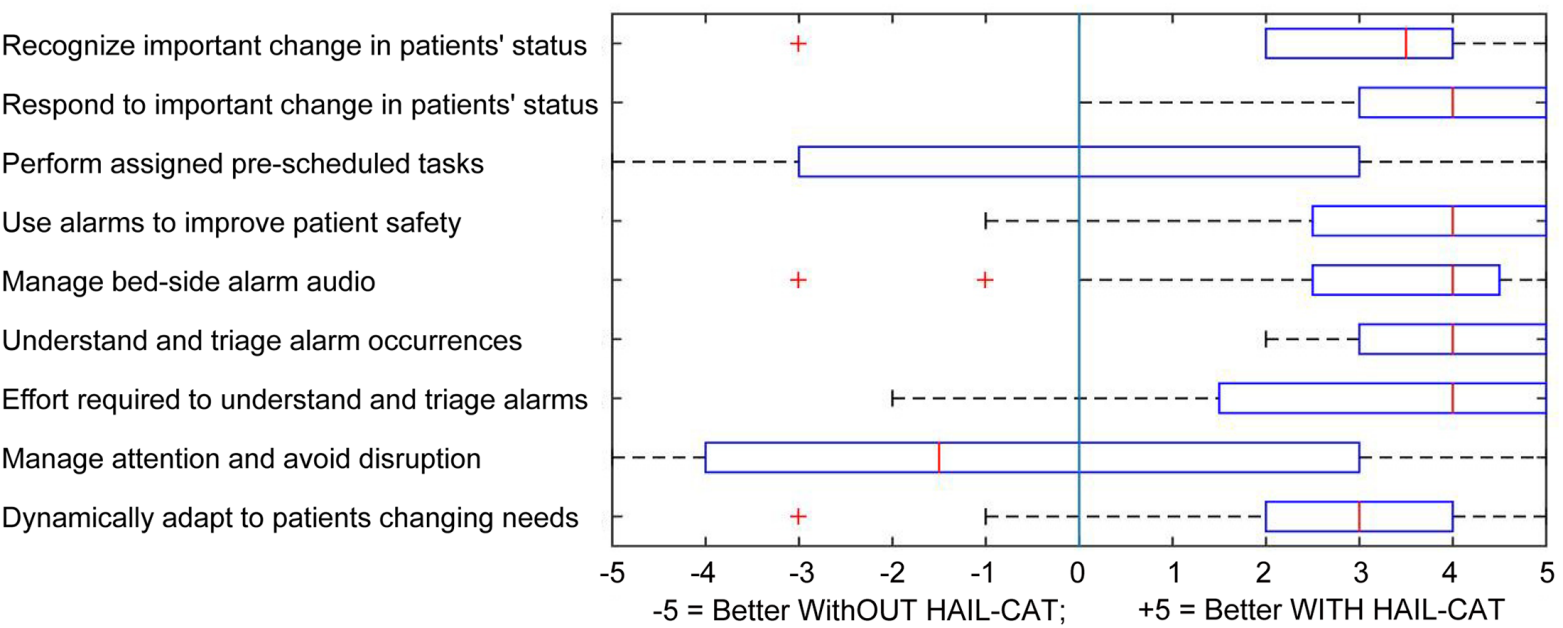
Nurses’ subjective opinions comparing their performance with and without HAIL-CAT.

**Fig 5:**
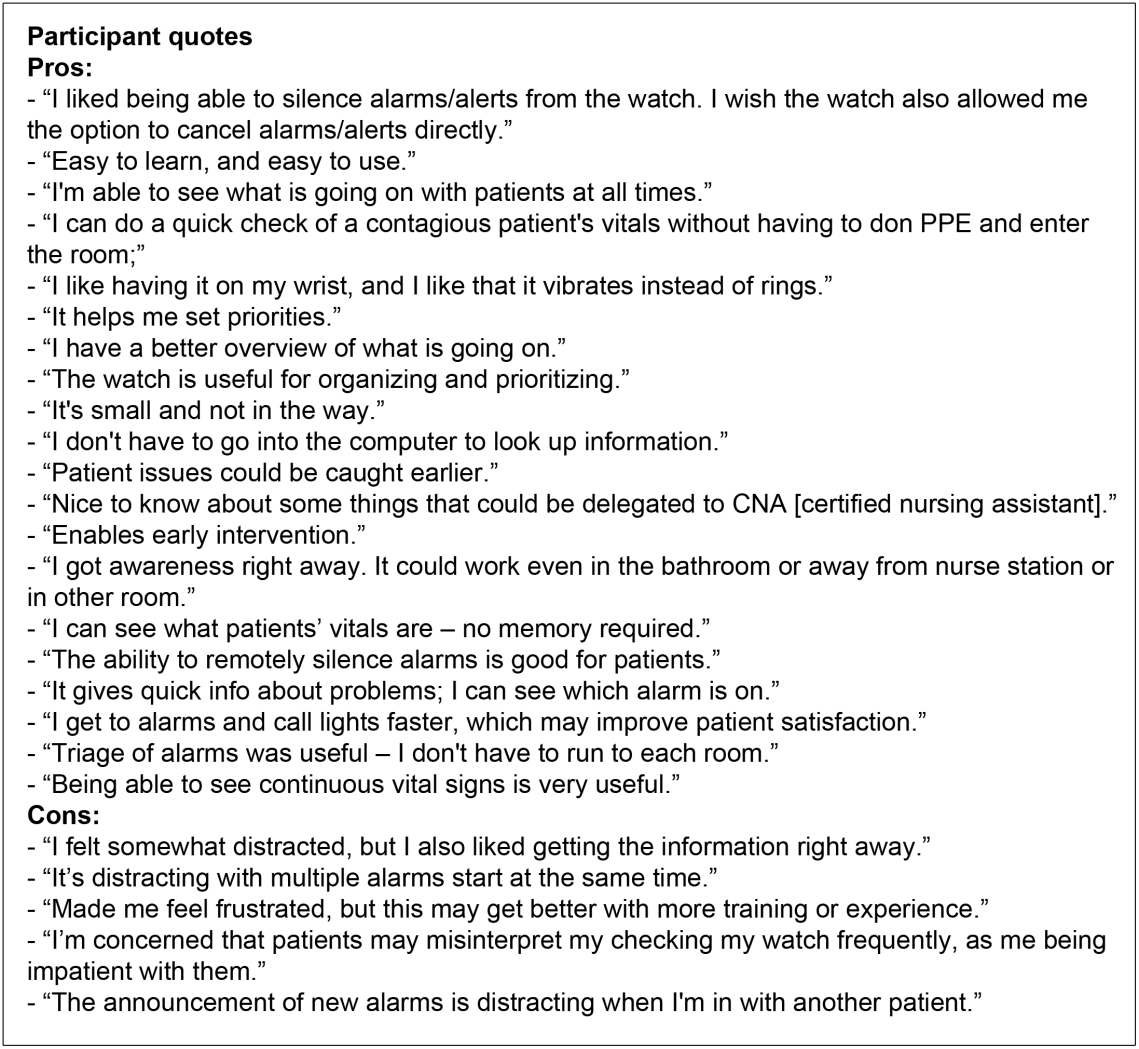
A sample of nurse quotes from a semi-structured exit interview about their impressions of using the HAIL-CAT aid wearable.

Fig 4 shows the results of an exit questionnaire that asked nurses to compare their performance with and without HAIL-CAT. A semi-structured exit interview was also conducted with each nurse (see Fig 5 for sampled quotes). Results show good consensus among nurses on preference for HAIL-CAT to support the recognition of clinically important changes or problems that signal onset of adverse events. Categories with highly favorable response for the introduction of HAIL-CAT include: recognize important change in patients’ status; respond to important change in patients’ status; use alarms to improve patient safety; manage bed-side alarm audio; and understand and triage alarm occurrences.

Nurses, however, disagreed about their relative feeling of disruption while using the prototype. It was observed that some nurses welcomed the alarm/alert notifications and felt supported in adapting their care delivery to match evolving needs. Other nurses, although they did not welcome being interrupted in general, still felt that the HAIL-CAT notifications were useful and would prefer to receive them anyway. There was also disagreement about the degree to which nurses felt that the introduction of the smartwatch affected their performance on their pre-scheduled care tasks. Some felt they did better with the smartwatch and others felt they did worse. The experimental observers who shadowed each nurse recorded the total amount of time that was required to complete all pre-scheduled tasks. They found no difference between control and treatment conditions. Subjective reports of difference in performance on pre-scheduled tasks were concluded to be limited to nurse perception with no observed actual effect on patient care in either direction.

In exit interviews, nurses said the wearable attention aid was also useful in coordinating delegation tasking of the nursing assistants. A policy for the experiment limited nurses to only be able to delegate response to patient call-light events to nursing assistants. Observers noted that when nurses received a notice of a call-light event on their smartwatches, that they would frequently get the attention of a nursing assistant and ask them to go respond. Nurses commented in exit interviews that this was useful, and that it would have been additionally useful to be able to delegate more kinds of tasks to nursing assistants. The utility of nurse delegation to care aids is recognized in other works [137].

Experimental observers noted strong individual differences among nurses. This agrees with other published work that highlights large individual differences across clinical staff [138]. Without the wearable attention aid, a large difference was noted regarding the degree of rounding. Some nurses rounded continually, and said they felt anxious not knowing how their patients were doing. Other nurses, when they completed their pre-planned tasks, stopped rounding. When the HAIL-CAT smartwatch was present, observations confirm the objective finding that consistency across nurses was improved for patient visits. Nurses who had continually rounded without the smartwatch were seen to pause in the hallway and use the smartwatch to check on all their patients. Then seeing them all stable, they would take a break from rounding. Other nurses who had gone on break without the smartwatch, would continue responding to alarms/alerts on their smartwatches even after having completed their pre-planned tasks.

There seemed to be variation across nurses in the degree to which they felt responsible to proactively act to maintain awareness of patients’ changing statuses. All nurses seemed committed to respond to important changes in their patients. However, in the control condition not all nurses would continually round across patients when there was no pre-scheduled task assignment. The HAIL-CAT notifications brought better situational awareness to all nurses and more consistency in patient visits across nurses.

## Discussion

Introduction of a wearable attention aid empowered RNs to respond at the bedside 148% faster on average to important clinically-actionable alarms. This breakthrough advance shows the high-potential for the general approach of empowering clinicians to more easily use existing alarms/alerts. Metacognitive technologies can support nurse attention management during high rates of alarms/alerts. This can enable them to more quickly recognize important changes and intervene sooner to preempt adverse events. Introduction of the HAIL-CAT wearable attention aid required almost no training, and no additional nurse staffing labor costs. This scope of practical improvement in addressing adverse events shows the potential of this approach to improve the financial predictability of patient care and allow outcome-based healthcare management.

By minimizing the workload for checking alarms, HAIL-CAT enables clinicians to access the currently dormant utility of multiple sophisticated alarm/alert generation systems that are already deployed. Analysis for this study is limited to describing improvement in response time to begin intervention in the onset of adverse events. It does not include an assessment of the precise degree to which the observed 148% gain would translate into percent reduction in total adverse events and financial consistency. A detailed comparison of these factors would require baseline information which is only estimated in the literature and producing it was outside of the scope of this study.

The occurrence of a new alarm/alert is a recommendation from a device (configured per hospital policy) that an additional clinical visit for a patient be immediately incorporated into a clinician’s internal multitasking schedule. HAIL-CAT attempts to minimize the meta-level work for nurses by allowing them to triage these recommendations and coordinate how and when (or whether) they will affect patients’ care plans. Minimizing this meta-level work is crucial because nurses are busy and have few resources cognitive available to do anything beyond their multitasking of delivering care per the current care plan [33,132,139,140]. This together with cognitive science on the types and limits of human cognitive resources [141], explain the challenge nurses have in managing attention in an environment where their multitasking is perpetually interrupted [131,134,130,133].

Triage of a new alarm/alert event is a two-step cognitive process for clinicians. First, a clinician must expend effort and time to check the meaning of the alarm/alert event with sufficient context to understand its likely importance for the related patient. And second, she/he must then revise their internal multitasking plan to allocate sufficient time and other resources to respond appropriately to the alarm/alert. Both steps balance a trade-off between: the potential utility of a new alarm/alert, and the potential negative disruptive effects of an interruption to his/her set of other on-going multitasking across multiple patients [31,142,30]. The first step is especially difficult because the potential utility of the new alarm/alert is still unknown. "Should I spend time and cognitive effort checking on the meaning of this new alarm, given the fact that that interruption will disrupt my important on-going tasks?" If the time and effort required for the first step is high, and the rate of non-actionable or false alarms is also high, then clinicians have a problem accepting highly-probable distraction when they are already doing important work.

In this light, the existing alarm safety crisis is not just too many alarms. A core problem is that existing alarm/alert notification solutions require too much time/effort of nurses to check their relative meaning and actionability. Solely by minimizing the work of checking alarms, HAIL-CAT improves the value of every alarm/alert to such a degree that nurses are able to check and triage every alarm/alert. At current rates in hospital, an acute care nurse with five patients will receive about 20 alarms/alerts per hour [59]. If the work of checking each alarm is a minute or more of work, nurses are being asked to spend one third of their total time checking alarms. Analysis of experimental video sample data show that the HAIL-CAT attention aid enables nurses to triage new alarms in 2–3 seconds on average. This result was also confirmed by the experimental observers. With the work of triaging alarms/alerts minimized, it was observed that nurses can/will quickly sift through the ~90% of non-actionable alarms/alerts to see and do something about the important ~10%.

The HAIL-CAT approach integrates alarms/alerts from all different medical devices. The prototype notification system also delivers an information package with every alarm/alert that includes summary patient context data. This information package, together with the additional patient context data that is only available inside the clinician’s head, enables RN users to triage new alarms/alerts easily enough to afford improved response at the bedside when it is important. This success of the HAIL-CAT wearable attention aid prototype functionality, allows a unit to maintain high performance on the CMS-emphasized ’communications and care coordination’ work, without requiring any additional expensive clinical labor.

Nurses did not receive training on strategies for using the wearable attention aid. Instead, they were instructed to focus on their clinical role in the simulation scenario. They had the freedom to individually invent their own use cases and strategies for integrating the smartwatch into their normal workflows (or not). Experimental observers noted two primary use cases employed by most participants. First, nurses would react to incoming alarm/alert events (vibration of the wearable) by glancing down at the smartwatch briefly to check the alarm/alert information. Later in exit interviews, nurses reported using these glances to triage alarms/alerts and decide how/whether they would respond. Second, after completing some care delivery tasking and leaving a patient’s room, nurses would often stop in the hallway just outside the patient room they just left and bring up their arm to begin using the watch (with no corresponding alarm/alert event). Nurses would manually access the status screens for each of their five patients and check their vital signs. Both use cases provided nurses with information that they used to dynamically decide what to do next.

### Implications, future work, and limitations

Future work is needed to integrate with technologies that reduce the frequency of non-actionable alarms/alerts. During exit interviews, nurses commented on the new opportunities provided by the smartwatch for improved delegation and potential remote cancelation of alarms. They also asked for configuration management and intelligent recommendation systems to improve safety [143]. It would be useful to investigate whether the introduction of the wearable attention aid causes change to the unit communication and coordination workflows.

The potential for improved training to further increase performance using the wearable attention aid could be explored. For the experiment reported here, the four nurses for each session received 10 minutes of training as a group prior on the technical operation of the smartwatch and its functions. However, to avoid biasing the experiment, nurses were not told anything about strategies for using the wearable attention aid, or that the primary metric for the study would be their time to respond at the bedside to important alarms. Nurses were left to individually invent their own use cases and strategies for using the smartwatch. In future work, perhaps after some initial trials using the smartwatch, nurses could receive some feedback on their performance. They could then be encouraged to discuss among themselves their ideas and experiences for using the smartwatch, and/or additionally receive training on strategies for use.

The within-subjects controls gave enough statistical power for conclusive hypothesis testing with 16 nurse participants. External validity was improved by nurses showing a great variety of clinical workflow strategies and performance. To further improve external validity, future research is needed for: multi-day testing; clinical trial with real patients; testing in other hospital environments; and additional exploration of infection control issues. Internal validity could be improved by comparison-testing the HAIL-CAT attention aid with other existing secondary alarm notification approaches. It is important to compare results across a set of different representative baselines beyond the "no secondary notification system" baseline used in this study. Additional baseline conditions could include: central station, pagers, wireless phones, and smart phones (without patient context data).

These results could be leveraged in a future work to create a detailed metacognitive model of the cost/benefit trade-off estimations that clinicians must do to triage alarm/alerts. Such a model could be used predictively to guide design of future secondary alarm notification systems.

## Conclusion

Improving nurses’ ability to quickly triage alarm/alert signals empowers them to strategically focus attention on clinically important changes while minimizing disruption of non-actionable interruptions. Effective dynamic allocation of the important resource of nurse attention, allows intervention at the bedside much sooner when it is most needed. Quicker action can prevent adverse events and their associated uncontrolled costs. This approach was tried to empirically explore its general potential for addressing the existing patient safety crisis related to alarms/alerts.

Innovative alarm mediation methods, proven in military combat systems, were leveraged to create a prototype wearable attention aid for nurses. This research facilitation, called HAIL-CAT, was introduced in highly-realistic 20-bed patient simulation of a full acute care hospital unit. The scenario included representative high rates of alarms/alerts (30 per each of two 90-minute scenario parts), including only 10% of alarms being important or clinically-actionable. Nurses received existing levels of alarms with no advanced filtering or alarm generation. Sixteen RNs participated in a randomized within-subjects single-factor clinical experiment. A control condition represented a hospital with no secondary alarm notification system. A treatment condition introduced use of the HAIL-CAT prototype.

The design of the experiment leveraged military R&D methods that balance internal and external validity. Every aspect of the experiment prioritized maximizing the clinical realism in representing a typical U.S. hospital acute care unit. Anything artificial that could potentially favor the results of the introduction of the wearable was considered a dangerous confound and systematically eliminated in pilot testing before the experiment was begun. At the beginning of the experiment, the introduction of the HAIL-CAT wearable attention aid prototype was explained to participants as just a minor part of the overall clinical experiment. Nurses were instructed to focus on delivering care to their assigned patients (simulated), and to either use or not use the smartwatch however they felt most appropriate. Actions to heighten realism included: clinically validated and realistic scenario; a complete 20-bed acute care unit simulation facility; minimal training for nurses on operation of the wearable attention aid; recruitment that emphasized the clinical scenario; randomized balance of treatment ordering; repeatability of the simulation; and experimental staff focused on clinical realism. Natural interaction among nurse participants during their various care delivery activities also enhanced the realism of the situation.

After the experiment, the methods chosen to analyze collected observation data were cautious to avoided issues related to assumptions of normal distributions or the possible influence of outliers. This innovative, yet conservative, experimental approach resulted in a statistically significant two-and-a-half-fold improvement in nurse response time on average to onset of possible adverse events. Evidence confirms the possibility that patient safety can be dramatically improved through introduction of metacognitive aids that help clinicians more easily triage existing alarm/alert signals. Additionally, simulation-based methods enabled repeatable high-fidelity quantitative experimentation and have demonstrated potential utility for future exploration of important healthcare concerns.

## Supporting information

S1 Table Experimental data. (XLSX)
